# Parasitic Infections and Carcinogenesis: Molecular Mechanisms, Immune Modulation, and Emerging Therapeutic Strategies

**DOI:** 10.32604/or.2025.071891

**Published:** 2026-01-19

**Authors:** Marta Pawłowska, Dorian Jarek, Jan Milanowski, Karolina Szewczyk-Golec

**Affiliations:** 1 Department of Medical Biology and Biochemistry, Faculty of Medicine, Ludwik Rydygier Collegium Medicum in Bydgoszcz, Nicolaus Copernicus University in Toruń, 24 Karłowicza St., Bydgoszcz, 85-092, Poland; 2 Student Research Club of Medical Biology and Biochemistry, Department of Medical Biology and Biochemistry, Faculty of Medicine, Ludwik Rydygier Collegium Medicum in Bydgoszcz, Nicolaus Copernicus University in Toruń, 24 Karłowicza St., Bydgoszcz, 85-092, Poland

**Keywords:** Cancer, carcinogenesis, parasites, pathogens

## Abstract

Parasitic infections are increasingly recognized as contributors to cancer development, yet the underlying oncogenic mechanisms remain insufficiently understood. Growing evidence from molecular oncology, immunology, and microbiome research suggests that chronic parasitic infections may drive tumorigenesis through sustained inflammation, deregulated signaling pathways, genomic instability, and the release of parasite-derived exosomes that reshape the tumor microenvironment. These insights underscore the need to integrate parasitology with cancer biology to understand infection-associated malignancies better. The aim of this narrative review is to synthesize current knowledge on how selected parasites contribute to cancer development and to highlight emerging therapeutic and diagnostic opportunities. We examine pathogens such as *Schistosoma haematobium*, *Opisthorchis viverrini*, *Toxoplasma gondii*, *Plasmodium falciparum*, and *Leishmania* spp., detailing their roles in chronic inflammation, immune modulation, and interactions with tumor-associated immune cells. The review further discusses parasite-induced immunosuppression, coinfections, and their cumulative impact on cancer risk. Additionally, we explore novel therapeutic approaches, including pathway inhibitors, epigenetic drugs, microbiome modulation, and engineered parasites. Future perspectives emphasize parasite-based immunotherapies, long-term epigenetic consequences of infection, and AI-driven multi-omics strategies for identifying oncogenic signatures. This review integrates advances from parasitology and oncology to provide new insights into biomarkers, targeted therapies, and mechanisms of infection-induced tumorigenesis. The literature search covered studies indexed in PubMed, Scopus, and Web of Science up to July 2025.

## 1 Introduction

Parasitic infections are increasingly recognized as significant contributors to cancer development. However, the precise mechanisms by which parasites promote oncogenesis remain underexplored [1]. Recent advances in molecular oncology and immunology have highlighted the complex interactions between parasites and host cellular processes that lead to cancer development and progression. Chronic inflammation, persistent activation of oncogenic signaling pathways, and genomic instability induced by parasite-secreted factors are now recognized as key players in cancer initiation and progression in infected individuals [1,2]. Although significant progress has been made in elucidating these mechanisms, much remains to be discovered, particularly regarding the molecular interactions between parasites and host cells [1,3]. Further research in this area is crucial for developing targeted interventions and refining cancer prevention strategies in populations at risk.

Exosomes and parasite-derived biomolecules appear to be key mediators in the interaction between parasitic pathogens and their hosts. They exert a profound influence on the tumor microenvironment, facilitating cancer progression [4,5]. These extracellular vesicles and secreted factors transfer a variety of proteins, lipids, and genetic materials, including microRNAs and other noncoding RNAs, from the parasite to host cells. In doing so, they modulate cell signaling and immune responses, as well as influence tissue architecture [6]. In particular, pathogens such as *Schistosoma haematobium*, *Opisthorchis viverrini*, *Toxoplasma gondii*, *Plasmodium falciparum*, and *Leishmania* spp. have been identified as key players in the etiology of specific human cancers [7,8]. Chronic inflammation, immune modulation, and direct tissue damage often mediate the oncogenic potential of parasites [3]. This information highlights the urgent need for a comprehensive understanding of parasite-induced carcinogenesis, especially in regions where parasitic infections are endemic and contribute significantly to the global cancer burden. A better understanding of these mechanisms could pave the way for the development of novel biomarkers for early cancer detection. It will also enable targeted therapies and effective public health interventions aimed at reducing the impact of infection-related cancers worldwide. Infection-attributable cancers remain a significant global burden, disproportionately affecting low- and middle-income countries. Recent syntheses estimate that infections account for roughly 15%–20% of all cancers in Low- and Middle-Income Countries (LMICs), with helminths representing the third leading group of oncogenic pathogens after viruses and bacteria. Endemic exposures drive distinct patterns. For example, *S. haematobium*–associated bladder squamous cell carcinoma (SCC) in sub-Saharan Africa and *Opisthorchiidae*-associated cholangiocarcinoma in Southeast Asia, underscoring the public-health importance of targeted prevention and control [2].

This narrative review aims to synthesize the current knowledge of the molecular and immunological mechanisms underlying parasite-induced carcinogenesis. In this paper, we emphasize the importance of emerging therapeutic strategies and propose future research directions. By connecting the fields of parasitology and oncology, this work aims to provide new insights into biomarkers, innovative therapies, and broader implications of the mechanisms of parasitically induced cancer for global health.

## 2 Methods

This narrative review was based on a structured literature search of PubMed (https://pubmed.ncbi.nlm.nih.gov/), Scopus (https://www.scopus.com/), and Web of Science (https://www.webofscience.com/wos/) databases. Articles published up to July 2025 were screened for relevance to parasitic infections, immune modulation, and cancer-related pathways. Preference was given to peer-reviewed studies, high-impact reviews, and original research. As this is a narrative review, no new experiments, reagents, or materials were used.

## 3 Molecular Mechanisms of Parasite-Induced Carcinogenesis

### 3.1 Chronic Inflammation and Oncogenic Signaling

Parasitoses are recognized as a significant factor contributing to the development of cancer, especially in regions where such infections are common. The molecular mechanisms underlying parasite-induced carcinogenesis most often involve chronic inflammation and sustained activation of signaling pathways associated with cancer [3]. These processes create conditions that favor tumor formation and growth. Many parasites, such as *S. haematobium*, *O. viverrini*, and *C. sinensis*, cause long-term infections that damage tissues repeatedly. As the body tries to repair this damage, inflammation persists in the affected organs [9,10]. In response to the parasites, immune cells such as macrophages, neutrophils, and lymphocytes continuously flock to the site of infection. These cells secrete inflammatory mediators, including cytokines such as interleukin-6 (IL-6) and tumor necrosis factor α (TNF-α), as well as reactive oxygen species (ROS) and reactive nitrogen species (RNS). These substances can cause DNA damage, promote mutations, and disrupt normal cell function. Another consequence of cyclical tissue damage and repair is the development of fibrosis and structural remodeling. These changes increase the risk of neoplastic transformation [3,11,12].

**Figure 1:**
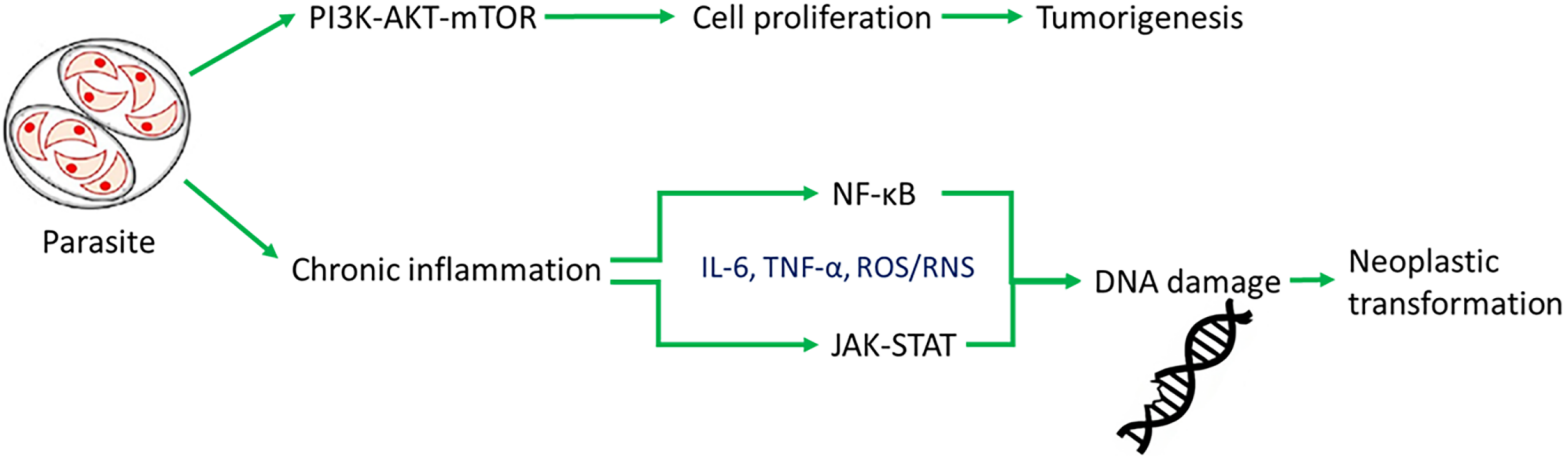
Host-parasite-cancer pathways. Schematic representation of the molecular signaling pathways activated by parasitic infection and their potential impact on neoplastic processes. The parasite induces chronic inflammation and activation of the nuclear factor kappa-light-chain-enhancer of activated B cells (NF-κB) and Janus kinase signal transducer and activator of transcription (JAK-STAT) pathways, leading to cytokine production and DNA damage. Prolonged activation of these mechanisms can promote neoplastic transformation. Simultaneously, the parasite activates the phosphoinositide 3-kinase/protein kinase B/mammalian target of rapamycin (PI3K-AKT-mTOR) pathway, which promotes cell proliferation and may lead to tumorigenesis. Green arrows indicate activation of immune pathways, while red arrows indicate signaling promoting tumor development

Chronic inflammation associated with parasitic infections activates several key signaling pathways that play a crucial role in the development of tumors. One of these is the nuclear factor kappa-light-chain-enhancer of activated B cells (NF-κB) pathway [13]. Persistent inflammatory stimulation leads to the activation of nuclear factor NF-κB in host cells (see Fig. 1). This factor regulates the expression of genes responsible for cell proliferation, survival, and the inflammatory response. This promotes resistance to apoptosis and facilitates processes that promote cancer cell survival (see Table 1) [14]. For example, *Cryptosporidium parvum* infection activates NF-κB and induces antiapoptotic signaling, which can lead to the neoplastic transformation of epithelial cells [15]. Another pathway is the Janus kinase/signal transducer and activator of transcription (JAK/STAT) pathway. Cytokines produced during chronic inflammation, particularly IL-6, activate Janus kinase (JAK) and STAT proteins. Activation of STAT3 is associated with increased cell proliferation, inhibition of apoptosis, and increased angiogenesis, which are hallmarks of cancer (see Table 1) [16]. The phosphoinositide 3-kinase/protein kinase B/mammalian target of rapamycin (PI3K/AKT/mTOR) pathway is equally essential. Growth factors and cytokines can activate phosphatidylinositol 3-kinase (PI3K), which in turn activates the AKT and mTOR proteins (see Fig. 1). For instance, *S. haematobium* antigens stimulate IL-6-mediated activation of JAK/STAT3 signaling in urothelial cells. Similarly, *O. viverrini* promotes NF-κB activation in bile duct epithelium via chronic inflammation. This pathway promotes cell growth, survival, and metabolic changes. Its frequent activation is observed in inflammation-associated cancers (see Table 1) [17]. Parasitic infections can utilize this mechanism to support the survival and proliferation of both infected and transformed host cells [18]. Chronic inflammation and the activation of oncogenic signaling pathways such as NF-κB, JAK/STAT, and PI3K/AKT/mTOR are central to the molecular mechanisms by which parasites promote carcinogenesis [19,20]. These pathways, along with inflammation-driven immune modulation, are further discussed in Section 5.

**Table 1:** Key pathways in parasite-induced carcinogenesis

Pathway	Role in carcinogenesis	Example of parasite involvement
**NF-κB**	Enhances inflammation, cell survival, and resistance to apoptosis	*Cryptosporidium parvum*-induced NF-κB activation in epithelium
**JAK/STAT**	Drives proliferation, inhibits apoptosis, enhances angiogenesis	IL-6-mediated STAT3 activation during chronic helminth infection
**PI3K/AKT/mTOR**	Supports cell growth, survival, and metabolic reprogramming	Cytokine-driven PI3K/AKT/mTOR signaling in inflamed tissues

Note: Abbreviations: JAK/STAT—Janus kinase signal transducer and activator of transcription; NF-κB—nuclear factor kappa-light-chain-enhancer of activated B cells; PI3K/AKT/mTOR—phosphoinositide 3-kinase/protein kinase B/mammalian target of rapamycin; IL-6— interleukin 6.

### 3.2 Genomic Instability and DNA Damage

Parasitoses contribute to cancer development directly, inducing genomic instability and DNA damage in host cells (see Fig. 1). This means that parasites can harm the genetic material of host cells in more than one way. These effects are mainly due to factors secreted by the parasites, which generate oxidative stress (OS), promote mutagenesis, and induce epigenetic changes. Together, these effects increase the risk of cancer development [21,22]. Many parasites release excretory products that stimulate the production of ROS and RNS in host tissues. Elevated concentrations of these molecules lead to OS, which damages genetic material. This damage includes DNA strand breaks, nitrogenous base modifications such as 8-oxoguanine, and the formation of DNA-protein crosslinks. If such damage is not effectively repaired, it can lead to mutations, chromosomal aberrations, and genomic instability. Such changes are hallmarks of cancer initiation and progression [21,23].

Parasitic infections can also influence the epigenetic programming of host cells. One mechanism involves altering DNA methylation patterns. It can lead to the silencing of tumor suppressor genes or the activation of oncogenes. Chronic liver fluke infection, for example, is associated with hypermethylation of key regulatory genes in bile duct cells [24]. Furthermore, inflammatory factors and parasite products can modify histone structures by altering their acetylation and methylation, thereby affecting chromatin condensation and gene expression [25]. Some parasites also affect the levels of microRNAs and long non-coding RNAs in host cells, further disrupting the regulation of the cell cycle, apoptosis, and DNA repair [26].

Parasite-secreted factors play a central role in inducing genomic instability and DNA damage in infected tissues. By promoting OS, facilitating mutagenesis, and reprogramming the epigenetic landscape, parasites create a cellular environment that is highly susceptible to malignant transformation. These mechanisms highlight the multifaceted ways in which parasitic infections can contribute to carcinogenesis, extending beyond the role of inflammation alone.

### 3.3 Parasite-Derived Exosomes and Biomolecules

Parasite-derived exosomes are a type of extracellular vesicle that play a key role in modulating the tumor microenvironment and promoting tumor progression [27]. Both parasites and infected host cells secrete them. They act as carriers of oncogenic signals, microRNAs, cytokines, and other bioactive molecules [28,29]. These exosomes carry a variety of cargoes, including proteins, lipids, DNA, mRNA, microRNA, and cytokines. Parasite sources include *T. gondii*, *Leishmania* spp., and *S. haematobium*, whose vesicles have been shown to transport immunomodulatory proteins, miRNAs, and cytokines that activate STAT3, PI3K/AKT/mTOR, and NF-κB, fostering proliferation, survival, and immune escape [30–33]. Their contents can directly affect cells in the tumor microenvironment, altering their function and promoting carcinogenesis [6,28]. By transferring their contents to host cells, parasitic exosomes are capable of reprogramming cellular pathways, inducing immunosuppression, and sustaining chronic inflammation, all of which promote tumor development. Parasites such as *T. gondii, Leishmania* spp., and *S. haematobium* have been shown to produce exosomes that carry oncogenic or immunomodulatory molecules. These vesicles can deliver oncogenic factors that activate key signaling pathways [28,29].

**Figure 2:**
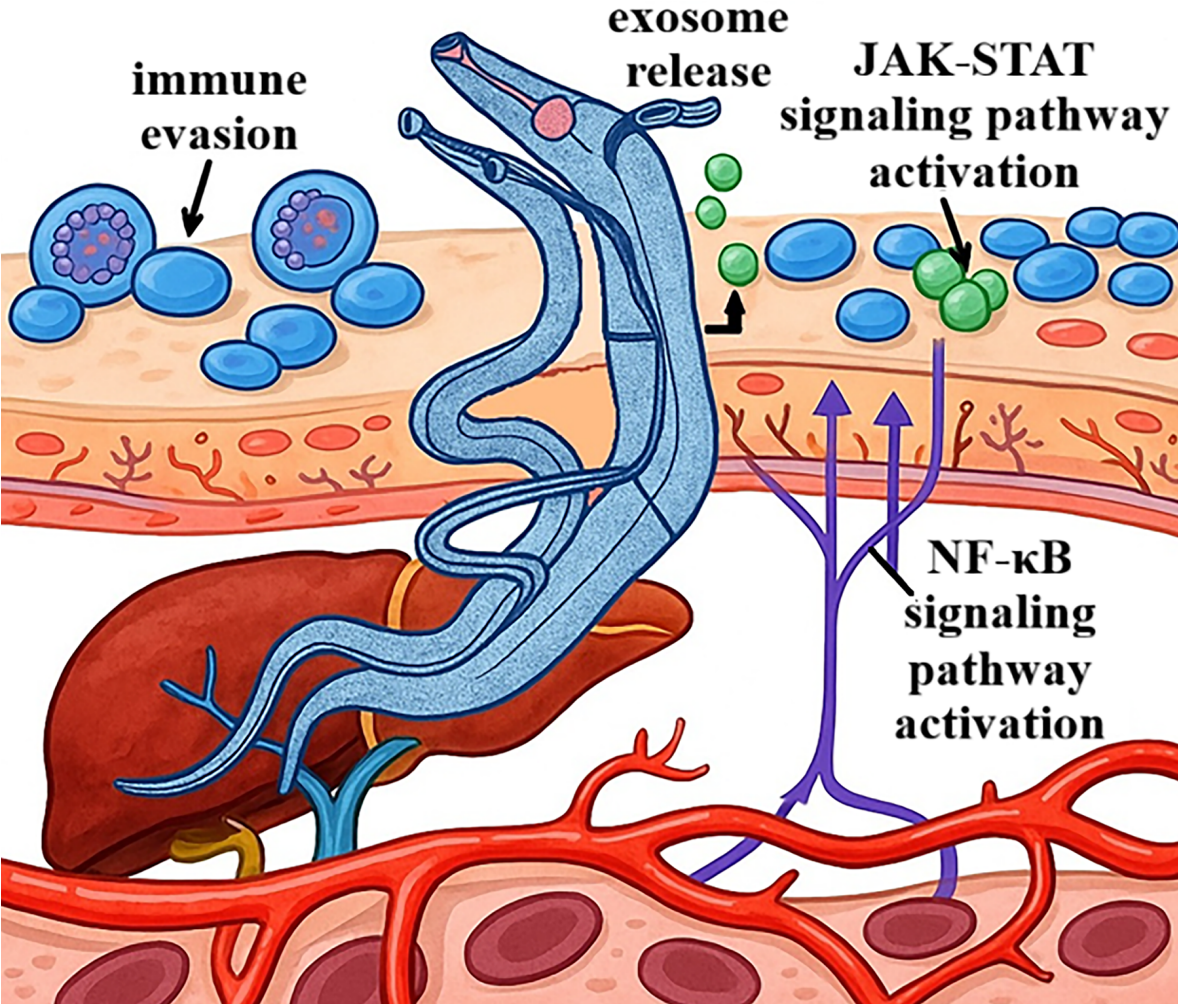
*Schistosoma* parasite migration and host interaction mechanisms. Schematic diagram of parasite-host interactions during infection. The parasite releases exosomes (exosome release), which modulate the host’s immune response by activating JAK-STAT and nuclear factor kappa-light-chain-enhancer of activated B cells (NF-κB) signaling pathways. Activation of these pathways leads to changes in the expression of genes associated with inflammatory and immune responses. Additionally, the parasite employs immune evasion mechanisms, allowing it to persist within the host and sustain infection over an extended period, including within the liver and blood vessels

At the same time, exosomes inhibit the activity of dendritic cells and natural killer cells. They also transport cytokines, such as IL-6 and granulocyte-macrophage colony-stimulating factor (GM-CSF) [30,31]. These cytokines exacerbate inflammation and promote tumor development. Furthermore, microRNAs contained within exosomes can silence tumor suppressor genes or activate oncogenes in target cells, thereby contributing to genomic instability and the progression of neoplastic transformation [32]. An example of the action of parasite-derived exosomes is those derived from dendritic cells infected with *T. gondii*. In experimental models, these exosomes have demonstrated the ability to modulate immune cell populations and inhibit tumor growth [33]. Exosomes from various sources, including parasites, are now recognized as essential elements shaping the tumor microenvironment, promoting angiogenesis, metastasis, and immune evasion, as well as potential biomarkers in the diagnosis and prognosis of cancer [34]. Given their molecular cargo, parasite-derived exosomes are being explored as potential biomarkers for early detection of parasite-associated malignancies, offering a non-invasive diagnostic tool. Schematic representation showing how parasites release exosomes that interact with host immune and epithelial cells, promoting JAK-STAT and NF-κB pathway activation and facilitating immune evasion (see Fig. 2).

### 3.4 Host Metabolic Reprogramming

Parasitic infections can significantly alter host metabolism by creating a microenvironment conducive to cancer initiation and development. One crucial effect of parasitic infection is a metabolic shift in host cells, from oxidative phosphorylation to aerobic glycolysis, known as the Warburg effect [35]. This reprogramming occurs even in the presence of oxygen, leading to increased glucose uptake and lactate production. This provides cells with the energy necessary for rapid proliferation and survival in unfavorable conditions. Lactate accumulation acidifies the tumor microenvironment, facilitating immune evasion and promoting angiogenesis [36].

Parasites also affect the metabolism of immune cells, such as macrophages and T lymphocytes, that infiltrate infected tissues. These changes promote the development of an immunosuppressive phenotype, which weakens the host organism’s ability to fight cancer cells and perpetuates chronic inflammation, supporting tumor growth. In response to infection, both parasites and host cells produce metabolites that can act as oncometabolites. These include ROS and specific amino acids. These compounds promote DNA damage, induce epigenetic changes, and support the survival of cancer cells [1,37,38].

Another important aspect is the impact of parasites on the intestinal microbiota. Infections can disrupt the normal balance of gut bacteria, changing how bacterial metabolites are produced. These shifts influence the host’s metabolism and immune response. As a result, parasite-microbiota interactions may create an environment that supports tumor growth by altering inflammatory and metabolic processes [39]. *T. cruzi* infection has been shown to alter host cell metabolism. This parasite influences the cell cycle, glycosylation processes, and metabolic pathways, which may contribute to creating an environment conducive to cancer development [38]. Helminths with carcinogenic properties produce metabolites that lead to the formation of DNA adducts, further linking metabolic changes to mutagenesis and an increased risk of cancer development [40]. Parasites also affect the intestinal microbiota, and their modulation influences both metabolic and immunological processes in the host. These changes may have significant implications for cancer susceptibility and progression rates [41,42].

Parasite-induced metabolic reprogramming is a multifaceted process that promotes cancer development. By altering host cell metabolism, producing oncometabolites, acting on immune cells, and modifying the microbiota, parasites create conditions conducive to cancer initiation and progression. Understanding these metabolic interactions offers new avenues for cancer prevention and therapy in populations affected by parasitic diseases.

## 4 Notable Parasitic Pathogens and Associated Cancers

Certain parasitic infections are recognized as significant risk factors for the development of specific human cancers. The mechanisms often involve chronic inflammation, immune modulation, and direct tissue damage caused by the parasites.

### 4.1 Schistosoma haematobium

Several blood flukes, including *S. haematobium*, *S. mansoni*, and *S. japonicum*, cause schistosomiasis. The disease’s alternative name is bilharziosis or bilharziasis [43]. Its connection to squamous cell carcinoma (SCC) was first described in the World Health Organization’s International Agency for Research on Cancer (IARC) monography [44]. While multiple species cause disease, *S. haematobium* is the only species classified as a Group 1 carcinogen by IARC due to its strong association with bladder cancer. This parasite infects humans percutaneously and resides in the blood vessels of the pelvic cavity, primarily in the veins draining the bladder and genital tract. Once the worms are there, they mature and start reproducing. Female worms distribute up to 3000 parasite eggs into the circulation daily. Approximately half of the eggs reach the urinary tract, where they are excreted via urine, facilitating transmission to a new host. The remaining eggs may become trapped in tissues, contributing to chronic inflammation and carcinogenesis. The rest essentially accumulates in capillaries, causing fibrosis and granulomas, which, along with other schistosomiasis-related effects, lead to carcinogenesis [45,46]. *S. haematobium* promotes SCC in various ways, including through lysates of adult forms and eggs, which directly stimulate oncogenesis by releasing estrogen-like substances (see Table 2). These metabolites appear to play a significant role in promoting cancer, as they can directly cause mutations in proto-oncogenes and suppressor genes, such as TP53, Bcl-2, and KRAS, through the formation of covalent bonds and/or OS. Another cause of DNA alterations in bilharziosis is suppression of leukocytes, such as upregulation of the T-helper 2 population (relative to the T-helper 1 population), inhibition of cytotoxic immune response, and a significant amount of Tregs and B cells, all of which are caused by *S. haematobium* eggs [47]. That immune dysregulation, accompanied by alterations in tumor suppressor genes, may lead to further mutations [45,48]. Apart from that, a study by Mbanefo et al. [49] corroborated previous findings and described a parasite-derived host immunomodulatory protein (IPSE) in SCC pathogenesis, as well as an ortholog of an interleukin-4-inducing principle. IPSE, as one of the proteins secreted by *S. haematobium* eggs, promotes angiogenesis and cell proliferation [49]. With all of these mechanisms, *S. haematobium* is considered an essential carcinogen in the pathogenesis of SCC.

**Table 2:** Summary of parasites and related cancers

Parasite	IARC status	Oncogenic mechanisms	Type of cancer	Additional comments
***Schistosoma haematobium***	Group 1	Estrogen-like metabolites, TGF-β, IPSE, immunosuppression	Bladder SCC	High incidence in sub-Saharan Africa
***Opisthorchis viverrini***	Group 1	Secretory proteins, nitrosamines, chronic inflammation	Bile duct cancer	Related to diet (raw fish)
***Clonorchis sinensis***	Group 1	Parasite secretions, chronic inflammation, oxidative stress	Bile duct cancer	Present mainly in East Asia
***Toxoplasma gondii***	Not classified	miRNA regulation, cell cycle modulation, apoptosis inhibition	Gliomas, solid tumors	Frequent latent infection (30%–50% of the population)
***Plasmodium falciparum***	Group 2A	Immunosuppression, coinfection with EBV, hemin transposition	Burkitt’s lymphoma	Most common in children in tropical Africa
***Leishmania* spp.**	Not classified	Chronic inflammation, DNA methylation, scarring	Skin cancers, BCC, SCC	Often, a difficult differential diagnosis with cancer

Note: Abbreviations: BCC—basal cell carcinoma; EBV—Epstein-Barr Virus; IPSE—parasite-derived host immunomodulatory protein; SCC—squamous cell carcinoma; TGF-β—transforming growth factor β.

### 4.2 Opisthorchis viverrini and Clonorchis sinensis

Another notable parasitosis with a proven effect on carcinogenesis is opisthorchiasis. The liver fluke, *Opisthorchis viverrini*, was first described by Jules Poirier [8] in 1886 under the name *Distomum viverrini*. Its connections to oncogenesis were observed as early as 1955 [50], which ultimately led to *O. viverrini* being classified as a Group 1 carcinogen for cholangiocarcinoma during an evaluation by the IARC in 2009 [51]. Along with *O. viverrini*, there was another parasite classified as carcinogenic, *Clonorchis sinensis* (discovered in 1875) [52,53], which exhibits similar mechanisms of promoting carcinogenesis [51,54,55]. Parasite infects humans (or other mammals) through the eating of raw or undercooked fish containing its metacercaria. Worm’s intermediate form resides in intra- or extrahepatic bile ducts, previously invading them through the duodenum. Once inside, they grow to mature forms and feast upon biliary cells, causing chronic inflammation and carcinogenesis through various mechanisms [56,57]. The most direct effect is induced by the fluke’s feeding and movement, which leads to mechanical damage to the epithelium of the bile ducts. That, in turn, initiates the inflammatory process and promotes cell proliferation, which precedes ductal fibrosis, epithelial desquamation, and adenomatous hyperplasia, all of which contribute to the development of cholangiocarcinoma [54]. In effect, bile ducts may start clogging with excess tissue and the bodies of flukes, ultimately leading to successive dilatation and an increase in permeability of the ductulus [58]. This is accompanied by a drastically higher risk of bacterial coinfections, with a notable example being recurrent pyogenic cholangitis [59]. Another mechanism of carcinogenesis is linked to the secretions of trematodes. Key trematode secretions implicated in cholangiocarcinogenesis include Ov-GRN-1 (granulin-like growth factor), thioredoxins, and cathepsins (F, B1), which promote epithelial proliferation, inhibit apoptosis, and remodel basement membranes, respectively.

Trematoda secretions lead to mixed effects on inflammation, as they activate T cells and hasten the development of dendritic cells, upregulate toll-like receptors, and generate IL-6 and IL-8 as pro-inflammatory agents, while also producing IL-10 and TGF-β (see Table 2). That entire process ultimately results in a disbalance that favors the inflammatory response, which contributes to oxidative damage and mutations, crucial elements in cholangiocarcinoma development. Another carcinogenic effect of the flukes is associated with the production of higher levels of exogenous and endogenous nitrosamines in food containing the parasite and the carcinogen produced by its host in response to infestation [60]. Furthermore, adult worms secrete agents that inhibit apoptosis and promote proliferation, such as thioredoxins and the granulin-like growth factor Ov-GRN-1. Lastly, through cathepsins F and B1, *O. viverrini* destabilizes the basement membrane, facilitating further invasion of cholangiocarcinoma and promoting easier metastasis [58]. With these mechanisms explaining the carcinogenic influence of *O. viverrini*, there is evidence for a reduced prominence of cholangiocarcinoma, which is typically parasite-associated, through education and prevention of Trematoda infection [61,62]. All of these mechanisms included, opisthochoriasis makes up a strong factor of carcinogenesis of cholangiocarcinoma, making it more frequent, invasive, metastatic, and less likely to have a good prognosis.

### 4.3 Toxoplasma gondii

Next in line for oncogenic ability is *Toxoplasma gondii*, a parasite first described in 1908 by two separate teams, Charles Nicolle [63], who named it, and Splendore [64]. Both initially thought it to be another kind of *Leishmania*, but it was later proved to be a wholly different species. From then on, its grasp on humanity was largely elucidated, with Flegr et al. [65] suggesting that *T. gondii* resides in 30%–50% of humans. With that in mind, we’ll delve into the possible carcinogenic effects this parasite induces on a third to half of our worldwide population. While not openly regarded as a carcinogen, reports are showing a higher incidence of various tumors in areas with a more common prevalence of *T. gondii* [25–31], which is why it is thought to cause them, probably through the side effects of its attempt to survive in human tissues, notably the brain. The most direct of these is neuroinflammation, a reaction triggered by the mere presence of bradyzoites and cysts during chronic infection. Furthermore, there seems to be more to that—studies by Zeiner et al. [66] and Cannella et al. [67] suggest that *T. gondii* can alter levels of miRNA, non-coding strands of RNA that are responsible for gene expression. *T. gondii* can interfere with host microRNA expression by modulating transcription factors and epigenetic regulators. For example, it can suppress tumor suppressor miRNAs or upregulate miRNAs involved in immune evasion and proliferation, such as miR-21 and miR-155. This results in the development of dysregulated cytokine production and an excessive immune response (see Table 2). Another possible way to tamper with oncogenicity is through the genetic and epigenetic modification of the expression of proto-oncogenes (e.g., c-myc), genes responsible for DNA repair, and tumor suppressor genes (e.g., p53) [68–70]. Additionally, *T. gondii* appears to modify the cells’ ability to participate in programmed cell death, possibly to ensure the survival of the intracellular parasite. This is achieved through both extrinsic and intrinsic pathways by inhibiting various caspases and other apoptosis-related factors [68,70,71]. Additionally, studies indicate that the effect may be opposite for immune cells, such as CD4+ and CD8+ lymphocytes, and Natural Killer (NK) cells. The next notable contribution to carcinogenesis is made through alterations to the cell cycle, where the parasite forces the cell into phase S of mitosis and then halts the process at G2 or M [70]. With all of that in the equation, it seems that toxoplasmosis may constitute a carcinogen. However, it is essential to note that current findings have revealed varying effects on different malignancies. Research shows *T. gondii* infection has a strong correlation with various solid tumors, primarily in neuroepithelioma cell lines, but also others (eg, endocrine tumors, lung cancer, and other carcinomas). In contrast, it has a negative one with hematological malignancies (eg, leukemia, lymphomas, and other blood-based growths) [68]. There are also findings, described by Song et al. [72], that acute toxoplasmosis may suppress tumor growth, while chronic toxoplasmosis enhances this process, as measured by neoplasm size and weight. With many unknowns about *T. gondii’*s role in carcinogenesis, further research is undoubtedly needed to corroborate this initially proved and furtherly theorized causation.

Overall, while *T. gondii* appears to promote the development of several solid tumors, especially in neuroepithelial and endocrine tissues, some studies report an inverse relationship with hematologic malignancies such as lymphomas and leukemias. These discrepancies suggest a complex and possibly tissue-specific interaction that warrants further investigation. Mechanistically, *T. gondii* reprograms host non-coding RNA by altering transcription-factor activity and epigenetic regulators in infected cells, leading to up-regulation of oncomiRs (e.g., miR-21, miR-155) and suppression of tumor-suppressive miRNAs [66,67]. These shifts impact cytokine balance, DNA repair programs, and apoptosis control, aligning with observed pro-tumorigenic phenotypes in selected tissues [68–71].

Evidence remains heterogeneous: reports of positive associations with solid tumors contrast with inverse or null links for hematologic malignancies. Differences in study design, serologic vs. PCR ascertainment, and control for co-infections likely explain the divergence. We therefore frame *T. gondii* as a context-dependent modifier rather than a uniform carcinogenic exposure and highlight the need for standardized, longitudinal designs.

### 4.4 Plasmodium spp.

Among tumors, there is a particular phenomenon, springing from its connection to infections, called endemic Burkitt Lymphoma (eBL). eBL is a highly aggressive non-Hodgkin lymphoma, with exceptionally high prevalence in tropical Africa, being the most common childhood tumor in this part of the world [73–76]. With its peculiar epidemiology, this neoplasm gave rise to theories about potential vectors for the disease. Human Herpes Virus 4 (HHV-4), commonly known as Epstein-Barr Virus (EBV), quickly emerged as a key player in the pathology. However, with EBV having a widespread incidence around the globe, it couldn’t have been the sole factor in promoting eBL. That’s where parasitic infection comes to light, as malaria exists with high incidence in eBL’s endemic region, and that’s what led to a theory of *Plasmodium falciparum* (the deadliest human malaria-causing pathogen) coinfection with EBV as ground for eBL emergence [77,78]. These findings led to the recognition of *P. falciparum*, a parasite first discovered in 1880 by Alphonse Laveran [42], as a Group 2A (probable) carcinogen by the IARC in 2014 [79]. Primarily, the parasite’s impact on oncogenesis is believed to be mediated by the immunosuppression of T cells and NK cells, with concurrent activation of B cells. The immune inhibition causes a lower control of EBV-infected cells by inhibiting cell lysis, thereby restricting the lysis of cells infected by EBV, which leads to higher viremia of an already carcinogenic virus. This alone can promote eBL with minimal help (see Table 2) [77,80]. The parasite may promote EBV production through an alternative mechanism. Hemin, an oxidized byproduct released from affected erythrocytes, boosts viremia through lysis in B cell lines [80]. Also, through B cells, *P. falciparum* may contribute to eBL more directly. When activated by malignant malaria, there is a prominent elevation in immunoglobulin production by B cells. This process carries an occasional risk of oncogenic mutations caused by double-strand DNA breaks during B-cell maturation and development [73]. This may lead to the translocation of c-myc, a recognized and already mentioned proto-oncogene, to an unfortunate location near immunoglobulin loci [69,80]. While *P. falciparum* is already recognized as a probable carcinogen, researchers are actively seeking the causes of this specific effect caused by a single species within the *Plasmodium* genus. Epidemiological data also corroborate these findings, as eBL is highly prevalent in Sub-Saharan Africa and Papua New Guinea (endemic for *P. falciparum*), but not in countries such as India, where malaria is primarily caused by *P. vivax* [73]. A possible culprit may be *P. falciparum* erythrocyte membrane protein 1 (PfEMP1) or a relatively lower infection of erythrocytes by *P. vivax*; however, both require further study to definitively prove the significance of this difference in pathogenicity among the *Plasmodium* genus [73,80]. Moreover, even factors within the *P. falciparum* species that further the risk of eBL require deeper investigation, as a direct connection or particular serotypes don’t seem to shed more light on the more detailed causes [74]. Another interesting thing is that chronic malaria may be burdened with a higher than anticipated risk of eBL, as it is a result of an established, efficient immune response for severe acute malaria, leaving patients with chronic parasitosis without prominent symptoms [73,81]. Studies show that, taking all of this into account, further research on malignant malaria is needed to establish definitive mechanisms of eBL pathogenesis and potential therapeutic targets.

### 4.5 Leishmania spp.

Leishmaniasis, a disease caused by parasites from the genus *Leishmania*, appears to have some pro-cancer properties. These protozoans, first observed by Pyotr Borovsky [82], were later identified as the cause of the disease kala-azar (as it was known in India) independently by William Boog Leishman and Charles Donovan [83] in 1903 (hence the name of the most prominent species, *Leishmania donovani*). Since then, we have attributed to the parasite a concerning effect, namely the potential promotion of carcinogenesis [84]. First, with the pathogen causing visceral and cutaneous leishmaniasis, it warrants a differential diagnosis from cancer, which the parasitic lesions mimic [85,86]. Secondly, cases of actual malignancy have been observed to emerge from lesions, wounds, and scars left by the parasite, as well as parasitosis being recognized in patients with tumors (see Table 2) [87–89]. This situation raises the question: Which pathology was present first: the parasite or the cancer? A relationship between the protozoan and various skin tumors was observed, as well as other neoplasms [90]. A sufficient number of studies do not corroborate these findings. However, there seem to be some mechanisms through which *Leishmania* spp. should be considered a carcinogen, though not yet regarded as one. Chronic infection and inflammation, processes deeply described in other parasitoses in this review, are known to cause malignant modifications in affected tissues. Another mechanism already mentioned is the inhibition of apoptosis, which is also present in leishmaniasis, as it is an intracellular parasite. Similarities include dysregulation of gene expression (in this case, through DNA methylation), such as the inhibition of tumor suppressor genes, and oxidative stress, which affects both the parasite’s cells and infected cells, as well as healthy cells [90,91]. Healing and scarring processes caused by parasitic lesions promote cancer development by exposing deeply located, more fragile tissue to environmental factors, such as ultraviolet radiation, as well as starting cell proliferation for much-needed repair—that mechanism may lead to spiraling out of the immune system’s control and tissue becoming a tumor [90]. A significant factor contributing to the progression of leishmaniasis is the parasite’s tropism, which enables it to infect various cells, leading to a visceral variant of the disease and promoting the development of tumors, including basal cell carcinoma and squamous cell carcinoma, in the affected tissue [90,91]. Looking at the whole picture, *Leishmania* spp. looks like a culprit of promoting cancer. This is, however, not always supported in studies; for example, a study by dos Santos et al. [45] suggests that the protozoan may exhibit an ability to provoke an anti-tumor response, resulting in a better prognosis and prolonged survival. With limited knowledge on the subject, further research is needed to classify Leishmania as a carcinogen or to determine its role in contributing to malignancy.

Parasitic infections contribute to the global cancer burden, particularly in developing countries where these infections are more prevalent. Preventive measures, early diagnosis, and treatment of parasitic diseases are crucial strategies for reducing the incidence of associated cancers.

## 5 Immune System Interactions and Tumor Microenvironment

### 5.1 Parasite-Induced Immunosuppression

Building on Section 3.1, parasite-driven inflammation skews immunity toward regulatory and Th2-biased states that facilitate tumor immune evasion. Here, we focus on functional immunosuppression: Treg expansion, M2 polarization, programmed death-ligand 1 (PD-L1) up-modulation, and how these changes reshape anti-tumor surveillance rather than re-describing upstream NF-κB/JAK-STAT/PI3K activation. Many parasites promote the proliferation of Tregs [92]. However, in cancers, increased numbers of Treg cells in the tumor microenvironment lead to the suppression of the anti-tumor response, allowing cancer cells to proliferate uncontrolled. Such parasite-dependent Treg proliferation is a well-documented mechanism for immune evasion in both chronic infections and cancer [93,94]. Parasitic infections can induce increased PD-L1 expression directly or indirectly through changes in cytokine profiles and immune cell population composition. Furthermore, they enhance tumor immune evasion [95,96].

Moreover, parasites can secrete molecules that structurally and functionally mimic human cytokines [97]. At the same time, parasitic factors can inhibit the synthesis of proinflammatory and antitumor cytokines, such as interferon-γ (IFN-γ), which weakens the immune system’s ability to combat cancer cells [98]. By manipulating cytokine signaling, parasites create an immunosuppressive environment that favors both their survival and the development of tumors [99]. For example, some *Leishmania* species induce IL-10 production, which impairs effective antitumor responses. Helminth and protozoan parasites, in turn, demonstrate the ability to activate TGF-β pathways, which are also used by tumors to suppress immune attacks [100].

### 5.2 Interplay between Parasitic and Viral/Bacterial Coinfections

The tumor microenvironment is shaped not only by the tumor itself and the host immune system but also by the presence of various pathogens. Parasitic infections, particularly when combined with viral or bacterial agents, can act synergistically, disrupting immune regulation and promoting tumor development in multiple ways [101]. A clear example comes from equatorial Africa, where *P. falciparum* malaria and EBV often occur together. Their coexistence is strongly linked to endemic Burkitt’s lymphoma, the most common childhood cancer in this region. EBV infection alone is not enough to cause the disease, but repeated malaria infections make it much more likely [102]. Prolonged exposure to malaria leads to intense activation and proliferation of B lymphocytes, which increases the number of EBV-infected cells susceptible to malignant transformation. At the same time, malaria-induced immunosuppression weakens the body’s ability to control the viral infection, allowing for persistently high levels of EBV. Furthermore, *P. falciparum* infection can lead to excessive activation of the Activation-Induced Cytidine Deaminase (AID) enzyme in B cell germinal centers, thereby increasing the risk of c-myc gene translocation, a characteristic of Burkitt’s lymphoma. The combined effects of chronic immune stimulation, impaired viral control, and genetic instability create an environment conducive to the development of EBV-associated lymphoma [103–105].

Another example is infection with the liver fluke *O. viverrini*, which is a documented risk factor for cholangiocarcinoma. A growing body of research indicates that disturbances in the gut microbiota may further contribute to the development of this cancer [106]. Chronic infection with *O. viverrini* disrupts the composition of the gut and biliary microbiome, leading to the predominance of bacterial species with pro-inflammatory and carcinogenic effects. This type of dysbiosis promotes persistent inflammation, increases the production of carcinogenic metabolites such as secondary bile acids and nitrosamines, and weakens mucosal immunity. As a result, malignant transformation of biliary epithelial cells occurs [107]. The interplay of parasite-induced tissue damage, chronic inflammation, and altered microbiota enhances the process of carcinogenesis, exceeding the impact of the parasitic infection itself [108].

These examples demonstrate how parasites, viruses, bacteria, and the immune system can interact in complex ways to drive cancer formation and progression.

### 5.3 Impact on Tumor-Associated Macrophages and Myeloid-Derived Suppressor Cells

Parasitic infections can significantly alter the tumor microenvironment by altering the phenotype and function of myeloid cells, particularly tumor-associated macrophages (TAMs) and myeloid-derived suppressor cells (MDSCs) [109]. Macrophages present in the tumor microenvironment exhibit high plasticity and can polarize toward two main phenotypes. Classically activated M1 macrophages are proinflammatory and possess potent antitumor properties. They produce cytokines such as IL-12 and TNF-α and support cytotoxic T lymphocyte responses [110]. Alternatively, activated M2 macrophages participate in tissue repair, exert immunosuppressive effects, and promote tumor growth. They secrete anti-inflammatory cytokines such as IL-10 and TGF-β, support angiogenesis, and inhibit effective antitumor responses [111]. Parasitic infections often induce the conversion of macrophages from an M1 to an M2 phenotype within the tumor. This process is mediated by the action of parasitic molecules and an altered cytokine environment that favors the expression of M2-specific genes [112]. For example, helminth infections induce a strong Th2 and regulatory response, leading to the accumulation of M2 macrophages in the tumor microenvironment [113]. Protozoan parasites, such as *Leishmania* spp., can directly affect macrophage signaling pathways, promoting their polarization toward the M2 phenotype. M2 macrophages influence tumor development in several ways. First, they suppress the activity of cytotoxic T cells and promote the expansion of regulatory T cells, limiting immune surveillance. Second, they secrete factors that promote angiogenesis and extracellular matrix remodeling, which facilitate tumor growth and metastasis. Third, they create an anti-inflammatory environment that protects tumor cells from immune destruction [114,115].

In parallel with their effects on macrophages, parasitic infections can increase the recruitment and activation of MDSCs within the tumor. These cells are potent inhibitors of T cell responses and contribute to immune evasion through the production of arginase, RNS, and ROS. MDSCs often interact with M2 macrophages, creating a strongly immunosuppressive environment that further impairs the antitumor response and promotes tumor progression [116–118].

## 6 Emerging Therapeutic Targets and Translational Approaches

### 6.1 Targeting Parasite-Driven Oncogenic Pathways

Unless otherwise stated, most data derive from general oncology or preclinical parasite models. Parasite-specific clinical validation remains limited. Recent research has identified key molecular targets and novel therapeutic strategies that can disrupt these processes and reverse parasite-induced oncogenic changes [105,116,119]. For example, in an animal model of *O. viverrini-*induced *cholangiocarcinoma*, the use of an NF-κB inhibitor (bortezomib) led to a significant reduction in the number of precancerous foci and a decrease in the expression of IL-6 and TNF-α in liver tissue [120].

As mentioned above, one of the primary pathways activated by parasitic infections is the NF-κB pathway [20]. Inhibitors of this pathway, such as bortezomib and parthenolide, have demonstrated efficacy in preclinical models, reducing inflammation and inhibiting tumor growth. Their action may be significant in cancers associated with parasites, such as *Schistosoma* and *Opisthorchis* (see Table 3) [121,122].

**Table 3:** Therapeutic strategies targeting parasite-driven oncogenic pathways

Targeted pathway/Epigenetic change	Example inhibitors	Mechanism of action	Potential cancer types
**NF-** **κ** **B**	Bortezomib, parthenolide	Pro-inflammatory/anti-apoptotic signaling blocking	*Schistosoma*-, *Opisthorchis*-related cancers
**JAK/STAT**	Ruxolitinib, tofacitinib	Inhibition of cytokine-driven proliferation	Hematological, hepatobiliary cancers
**MAPK**	Trametinib, ERK inhibitors	Cell growth and survival signals disruption	Liver, bladder, and gastrointestinal cancers
**DNA methylation**	Azacitidine, decitabine	Gene silencing reversing; tumor suppressors restoring	Cholangiocarcinoma, bladder cancer
**Histone modification**	Vorinostat, romidepsin	Silenced genes reactivation; immunity-enhancing	Various parasite-associated cancers

Note: Abbreviations: ERK—Extracellular Signal-Regulated Kinase; MAPK—Mitogen-Activated Protein Kinase.

The JAK/STAT pathway is also frequently exploited by parasites to promote cell survival, proliferation, and immune evasion [123]. In a study on mice chronically infected with *S. japonicum*, administration of a JAK1/2 inhibitor (ruxolitinib) reduced the severity of bone fibrosis and limited epithelial cell proliferation, indicating the potential value of this class of drugs in preventing neoplastic transformation [124,125]. Inhibitors such as ruxolitinib and tofacitinib can block these signals, attenuating tumor-promoting inflammation and restoring immune surveillance. These compounds are currently being investigated for use in parasitic cancers, particularly hematologic and hepatobiliary cancers [126–128].

Another important pathway is the Mitogen-Activated Protein Kinase (MAPK) pathway, which regulates cellular responses to stress and inflammation [129]. MAPK inhibitors, such as trametinib, have been shown to reduce hepatocyte proliferation and inhibit angiogenesis in models of parasitic liver tumors. However, data are currently limited to preclinical studies [129–132]. The MAPK pathway is often overactivated in cancers associated with parasitic infections [133]. Inhibitors targeting components of this pathway, including trametinib and Extracellular Signal-Regulated Kinase (ERK) inhibitors, can disrupt abnormal proliferative and survival signals (see Table 3). Combining anti-MAPK therapies with immunotherapies may enhance the efficacy of treatment for parasitic-associated cancers [134–136].

Parasitic infections can also lead to epigenetic changes. Because these changes are reversible, they represent attractive therapeutic targets [137]. In animal models of cholangiocarcinoma caused by *O. viverrini*, the use of azacitidine led to the reactivation of tumor suppressor genes (p16INK4a) and reduced tumor proliferation [138]. DNA methyltransferase inhibitors, such as azacitidine and decitabine, restore regular gene expression and increase the susceptibility of cancer cells to immune elimination [139]. These compounds are currently being evaluated for their ability to reverse parasite-induced epigenetic changes, particularly in biliary tract and bladder cancer [140–142]. An additional therapeutic approach is modulating histone modifications. Histone deacetylase inhibitors, such as vorinostat and romidepsin, remodel chromatin structure and facilitate the reactivation of silenced genes while supporting an anti-cancer immune response [143–145]. Combining epigenetic therapies can lead to synergistic effects in restoring genetic and immune balance in host cells. However, it is essential to emphasize that most data on signaling inhibitors come from general cancer studies, not specifically from parasite-induced tumor models. This limits the ability to directly translate the results to endemic populations.

### 6.2 Exploiting Parasite-Derived Molecules for Cancer Therapy

Parasite-derived molecules, thanks to their unique immunomodulatory properties, are gaining increasing importance as innovative tools in cancer therapy. Promising research directions include the use of *T. gondii* antigens as immunological adjuvants and *Leishmania*-derived exosomes as cancer vaccine candidates [146,147].

*T. gondii* antigens exhibit potent immune-stimulating properties. They can break tumor-induced immune tolerance by activating dendritic cells and enhancing Th1 responses. This results in increased production of IFN-γ and enhanced cytotoxic T lymphocyte activity [148,149]. Preclinical studies in mouse models have demonstrated that the administration of *T. gondii* antigens, either in the form of live, attenuated parasites or antigen-pooled dendritic cell vaccines, significantly reduces tumor growth and volume. In studies on mice with colon tumors, immunization with *T. gondii* antigens (SAG1) resulted in a significant increase in CD8+ lymphocyte infiltration and a 40%–60% decrease in tumor volume [150,151]. Increased CD8+ cell infiltration, higher serum IFN-γ levels, and a shift in the tumor microenvironment toward an antitumor response were also observed. Even replication-deficient *T. gondii* strains and inactivated antigen preparations demonstrated the ability to reverse tumor-associated immunosuppression, further supporting their potential as safe adjuvants in cancer immunotherapy [152]. Their mechanism of action involves, among other things, the stimulation of antigen-presenting cells and the promotion of cross-presentation of tumor antigens, leading to the activation of specific T lymphocytes. Furthermore, these antigens influence myeloid cell populations within the tumor, reducing the number of immunosuppressive cells and promoting an effective antitumor response [153]. Currently, studies are underway to utilize *T. gondii* antigens as adjuvants in both prophylactic and therapeutic cancer vaccines [119,154].

Another innovative approach is *Leishmania*-derived exosomes. Naturally capable of delivering antigens to immune cells, *Leishmania* exosomes are being investigated as potential vaccine platforms that can induce both innate and adaptive immune responses [146]. In preclinical models, these exosomes have been demonstrated to elicit potent antigen-specific T-cell responses, promote dendritic cell maturation, and initiate cytotoxic reactions against tumor cells [155]. Advantages of exosome-based vaccines include their efficient antigen delivery, natural adjuvant properties, and the ability to tailor their content to specific tumor or parasite antigens [29,146]. *Leishmania major*-derived exosomes, used as tumor antigen carriers in a melanoma model, induced a Th1 response and reduced the number of lung metastases by 50% compared to the control [156]. Although clinical data are still limited, ongoing research is focused on optimizing the production, loading, and delivery of exosomes to maximize their anti-cancer efficacy [157].

The use of parasite-derived molecules, such as *T. gondii* antigens and *Leishmania* exosomes, opens a new perspective in cancer immunotherapy. It provides the opportunity to overcome the mechanisms of immune evasion by cancer cells and increase the effectiveness of anti-cancer vaccines. It’s worth distinguishing potential applications. Immunotherapy based on parasite antigens may play a role in both treating advanced tumors (by overcoming immunosuppression) and preventing relapse in at-risk patients in endemic regions. Despite promising preclinical results, significant challenges remain. These include ensuring safety and specificity of genetically modified parasites, as well as overcoming barriers to targeted delivery *in vivo*. Additionally, most studies stay in the experimental phase, with limited clinical translation.

### 6.3 Microbiome Modulation and Parasitic Therapy

As mentioned above in Section 2, the human gut microbiome plays a crucial role in maintaining immune balance and protecting against diseases, including cancer [158]. Parasitic infections can disrupt this delicate balance, leading to dysbiosis, a state of imbalanced microbiota that promotes inflammation and increases the risk of cancer [159]. A growing body of research suggests that modulating the microbiome, particularly through the use of probiotics and other nutritional interventions, can counteract the oncogenic effects of parasitic dysbiosis [160].

Parasites can influence microbiome metabolism by reducing the production of beneficial short-chain fatty acids and increasing the concentration of cancer-promoting metabolites, such as secondary bile acids [159]. Changes in the microbiome can also compromise gut barrier function, facilitating the entry of toxins and pathogens into the body and increasing inflammation and the risk of cancer development [161–163]. Supplementing with probiotics, which include beneficial bacteria such as *Lactobacillus* and *Bifidobacterium*, can help restore microbiome diversity, reduce inflammation, and support an immune response that is anti-cancer [164,165]. Li et al. [166] analyzed the impact of intestinal helminth infections on the development and treatment of colorectal cancer. The authors demonstrate that although helminth infections may reduce the risk of precancerous lesions and promote tumor progression through immunosuppression, their derivatives (antigens, exosomal miRNAs) demonstrate therapeutic potential. They may support immunotherapy and chemotherapy for colorectal cancer, particularly after the use of genetic engineering tools such as CRISPR to limit parasite pathogenicity [166]. Lee et al. [167] reported the first metagenomic study of humans infected with *Fasciola hepatica* in Peru, demonstrating specific features of the gut microbiome that distinguish infected individuals from healthy individuals. The authors concluded that differences in the composition and function of bacteria in treatment-responsive individuals and *Actinobacteria* in resistant individuals determine the efficacy of triclabendazole, suggesting the involvement of the microbiome in modulating treatment response and the potential for its use in predicting treatment outcome.

In mouse models infected with *S. mansoni*, the administration of probiotics (*Lactobacillus* spp.) resulted in a reduction of OS markers in the liver and intestine, as well as a decrease in the frequency of lesions in these organs [168,169]. Probiotic blends can mitigate dysbiosis caused by both parasites and anti-cancer treatments and also enhance the effectiveness of cancer therapies. Prebiotics, which are nutrients that feed beneficial bacteria, and symbiotics, which are a combination of probiotics and prebiotics, also support microbiome recovery and may reduce cancer risk [170]. Prebiotics and symbiotics are mainly studied in the context of prevention. Their action is based on the reconstruction of microbiota after parasitic infection and the reduction of chronic inflammation.

In animal models, probiotic supplementation has been shown to reduce tumor growth, lower inflammatory cytokine levels, and restore the typical composition of the gut microbiota [162,171]. In patients with colon cancer, postoperative probiotic administration has been shown to reduce gastrointestinal complications, increase microbiome diversity, and increase the number of beneficial bacteria [172]. Modulating the gut microbiome through probiotics, prebiotics, diet, and other interventions represents a promising strategy for mitigating the effects of parasitic dysbiosis and reducing the risk of cancer development. Ongoing research aims to refine these methods, which may enable their personalized and effective use in cancer prevention and treatment in the future.

### 6.4 CRISPR and Genetic Engineering Strategies

Genetic engineering, including CRISPR-based technologies, opens up new possibilities for using parasites as innovative tools in cancer therapy. Genetic modifications can transform parasites into safe and effective therapeutic agents that can overcome the limitations of traditional cancer treatments [173]. One area of research is the use of genetically modified parasites as oncolytic agents. These organisms are engineered to selectively infect and destroy cancer cells without damaging healthy tissue. They can directly lyse cancer cells or induce a strong immune response by releasing tumor antigens and activating the host’s immune system [5,174–176]. For example, a genetically modified *T. gondii* strain deficient in replication (cps1-1) in mouse ovarian cancer models led to complete tumor remission in 40% of animals. It significantly increased the survival of the remaining ones [72,177]. Additionally, these parasites can be programmed to produce therapeutic molecules or immune modulators, increasing their anticancer efficacy. Due to safety concerns, most studies focus on attenuated or replicative-deficient strains to minimize the risk of infection in patients [175,178]. Although clinical trials are still sparse, preclinical results suggest that genetically modified protozoa, particularly in combination with chemotherapy, may become a significant component of cancer therapy in the future. Amen et al. [179] described the application of CRISPR technology and antimicrobial peptides in combating antibiotic resistance and cancer, with particular emphasis on the role of the microbiome as a key element modulating immunity and homeostasis. The authors demonstrated that microbiome engineering using CRISPR enables the creation of probiotics that produce antimicrobial peptides with anticancer activity. This opens the door to personalized therapies and synergistic strategies against infections and cancer.

A second area of parasite use is the creation of cancer vaccine platforms. Modified parasites are capable of activating both innate and adaptive immune responses, transforming immunologically “cold” tumors into more treatable “hot” tumors [5,173,178]. Wei et al. [180] provided a detailed analysis of the mechanisms by which chronic pathogen infections remodel the tumor microenvironment. The authors emphasized that breakthrough therapeutic strategies include checkpoint inhibitors, cell therapies, vaccines, engineered bacteria, and microbiome modulation. However, the effectiveness of these approaches is limited by pathogen heterogeneity, lack of biomarkers, and the potential risk of reactivation.

Advances in genetic modification technologies, including the use of CRISPR, are significantly accelerating the development of parasite-based cancer therapies. Similar approaches, using parasites as vectors to deliver tumor antigens, are primarily used for prophylactic (vaccination) or supportive treatment of advanced tumors by activating the antitumor response. The creation of safe, precise, and effective oncolytic agents and vaccine platforms promises significant changes in cancer treatment in the coming years. Although promising, most studies on genetically modified parasites and CRISPR-based oncolytic vectors remain in the preclinical stage and require validation in human trials.

While all therapies described in Section 6 show promise in general oncology, their specific application to parasite-associated cancers remains largely theoretical. Experimental validation in parasite-specific tumor models is currently limited.

## 7 Future Directions and Unanswered Questions

Future research on the parasite-cancer relationship is heading in promising directions. One key area of investigation is whether parasite-based immunotherapies can enhance the efficacy of immune checkpoint inhibitors. Parasites have evolved numerous strategies to manipulate host immunity. This often leads to the suppression of the immune response, allowing the parasite to prolong its survival [181]. Some parasite molecules mimic or disrupt immune checkpoints, such as PD-1/PD-L1, while others activate Tregs, aiding tumor immune evasion [182,183]. Investigating whether parasite-derived antigens or genetically engineered parasites can be used to reprogram the tumor microenvironment, reduce immunosuppression, and synergize with checkpoint inhibitors could open new therapeutic avenues. Potential approaches include the use of *T. gondii* antigens as adjuvants to enhance the immune response against tumors. Another possibility is to develop vaccines or therapies based on *T. gondii-*derived exosomes to strengthen the immune system’s ability to fight cancer [184]. It is also worthwhile to conduct preclinical and clinical studies to determine whether combining parasite therapies with checkpoint inhibitors is a safe and effective approach.

Another important line of research is the long-term epigenetic consequences of chronic parasitic infections and their impact on cancer risk. Persistent inflammation and release of parasite-derived factors can lead to DNA methylation, modification of histone markers, and changes in noncoding RNA expression. Future research should focus on characterizing these epigenetic changes in tissues chronically exposed to parasites [137,185]. Long-term studies in endemic parasite populations could help track epigenetic modifications over time [91]. Functional studies are needed to determine whether these changes are reversible and directly contribute to carcinogenesis. Furthermore, exploring the potential of epigenetic therapies to counteract parasite-induced abnormalities could provide new strategies for cancer prevention or treatment.

A third important question is how changes in gut microbiota resulting from parasitic infections contribute to the development of cancer, also known as oncogenesis. Parasitic infections in the gastrointestinal tract often lead to disruption of the microbiota and dysbiosis. This, in turn, can promote chronic inflammation, immune dysregulation, and metabolic changes that promote cancer development. It is essential to elucidate the mechanisms by which these microbiota changes influence cancer risk, both locally (for example, in colorectal cancer) and systemically, through microbial metabolites and immune mediators [41,186,187]. Metagenomic and metabolomic profiling can be used to compare the gut microbiota of individuals infected with and those without the infection. Interventional studies using probiotics or microbiome-modulating therapies could assess their effectiveness in reducing the risk of cancer. Identifying specific microbial taxa or their metabolites that mediate the link between parasitic infection and oncogenesis could provide new diagnostic or therapeutic targets.

Ultimately, the application of AI-based multi-omics approaches has the potential to reveal novel parasite-driven oncogenic signatures. Given the complexity of host-parasite interactions and the multifactorial nature of cancer, integrative analysis encompassing genomics, transcriptomics, proteomics, and metabolomics is essential. Advanced AI models can be utilized to analyze large-scale, multi-omics datasets from parasite-infected tissues and cohorts of cancer patients, enabling the discovery of new biomarkers. Predictive models derived from such analyses can help differentiate patients according to their cancer risk [188,189]. This will allow more personalized and targeted prevention or treatment strategies. All these developments underscore the importance of interdisciplinary collaboration between parasitology, immunology, oncology, and computational biology to fully understand and reduce the risk of cancer associated with parasitic infections.

## 8 Conclusions

Parasitic infections are increasingly recognized as a factor contributing to cancer development, especially in endemic regions. Chronic infections induce long-term inflammation and activate signaling pathways that promote the survival of cancer cells, thereby weakening the immune response. Parasites can also damage DNA and influence epigenetic changes, leading to the activation of oncogenes. Furthermore, these infections disrupt the immune system, increasing the number of cells that suppress the antitumor response. Co-infections with other pathogens can exacerbate this effect. Moreover, parasites influence the tumor microenvironment, promoting its development.

New therapeutic strategies include drugs targeting activated pathways, parasitic substances as vaccine components, and modulation of the microbiome. Research on genetically modified parasites for therapeutic purposes is also promising. In the future, it would be worthwhile to focus on combining parasitic therapies with immunotherapy and analyzing the long-term epigenetic effects. An integrated approach using artificial intelligence can help develop more effective diagnostic and treatment methods.

Despite significant advancements, our understanding of parasite-derived carcinogenesis remains incomplete. Future studies should focus on mechanistic validation using *in vivo* models, evaluation of long-term co-infection outcomes, and development of parasite-specific therapeutic targets. Moreover, inconsistencies across cancer types and geographic patterns highlight the need for regionally tailored epidemiological surveillance.

Practically, our narrative review argues for: (i) region-tailored screening in endemic settings (e.g., urine-based hematuria screening plus ultrasound where *S. haematobium* is prevalent); (ii) integrating anti-parasitic control with cancer-prevention programs; and (iii) biomarker development (exosome cargo, epigenetic signatures) to identify high-risk, chronically infected individuals. These steps can translate mechanistic insights into measurable reductions of parasite-associated cancer burden.

## Data Availability

Not applicable.
